# A newly diagnosed case of polymorphous low-grade neuroepithelial tumor of the young 

**DOI:** 10.5414/NP301081

**Published:** 2018-04-27

**Authors:** Mireille Bitar, Shabbar F. Danish, Marc K. Rosenblum

**Affiliations:** 1 Department of Pathology,; 2 Department of Neurosurgery, Rutgers Robert Wood Johnson Medical School, New Brunswick, NJ,; 3 Department of Pathology, Memorial Sloan Kettering Cancer Center, New York, NY, USA

**Keywords:** polymorphous, low-grade, neuroepithelial, tumor

## Abstract

Polymorphous low-grade neuroepithelial tumor of the young (PLNTY) is a recently described variant of low-grade neuroepithelial tumors that exhibits infiltrative growth, histopathological variability with frequently prominent oligodendroglioma-like components, intense labeling for CD34, absence of 1P/19Q codeletion, a distinct DNA methylation signature and genetic alterations involving MAP kinase pathway constituents of either the B-Raf proto-oncogene BRAF or fibroblast growth factor receptors 2 or 3 (FGFR2 and FGFR3). We here report a newly diagnosed case of PLNTY involving the temporal lobe in a 31-year-old man with chronic focal epilepsy. This tumor had histologic and immunophenotypic features similar to the recently described PLNTY and proved BRAF V600E mutant. Biomolecular profiling is becoming increasingly important in characterizing neuroepithelial tumors. Furthermore, biomolecular features such as CD34 expression and BRAF mutation have been reported to be significantly associated with the clinical behavior of these tumors. Like other low-grade neuroepithelial tumors, PLNTYs appear to be generally indolent with excellent seizure relief after total surgical resection. It is important to recognize cases of PLNTY in order to guide clinical management including the indication for surgery.

## Introduction 

Low-grade neuroepithelial tumors encompass a group of central nervous system neoplasms characterized by a spectrum of glial or glioneuronal differentiation that manifest as early-onset pharmacoresistant epilepsy, accounting for more than half of the epileptogenic tumors in children and young adults [1, 2]. These tumors are classically represented by ganglioglioma and dysembryoplastic neuroepithelial tumor [1, 2, 3]. However, this group is expanding [3, 4] and has recently been found to include a distinctive entity designated as “polymorphous low-grade neuroepithelial tumor of the young” (PLNTY) [5]. 

Here, we report an example of PLNTY occurring in a 31-year-old man with a chronic refractory seizure disorder since childhood. The patient suffered complex partial seizures since age 10. Seizures were controlled by antiepileptic drugs but became refractory to treatment at age 17. At age 20, the patient underwent a partial temporal lobectomy and subsequently experienced less frequent seizures. Pathology material from his temporal lobectomy is not available for our review. However, he continued to have seizures as frequent as 12 times a month that would last for a few minutes, and include tight feeling in the chest, hand clenching, starring, drooling, and post-ictal confusion. Recent magnetic resonance imaging (MRI) with contrast showed a hypointense focus on T2-weighted images within the residual right temporal lobe posterior to the previous surgical cavity. 

Due to continued seizure activity, a revision right temporal lobectomy was offered to the patient. He underwent a therapeutic right temporal lobectomy including resection of residual hippocampus. Postsurgical MRI revealed complete resection of the temporal lobe structures. The patient recovered without complications and is free of seizures 12 months following the surgery. 

## Pathological examination and immunohistochemistry 

The tissue sections were embedded in paraffin, and histologic slides were stained with hematoxylin-eosin stain. Immunohistochemical studies were performed using a standard avidin-biotin immunoperoxidase technique and included the following antibodies; CD34 (QBEnd/10, Leica, Buffalo Grove, IL, USA), glial fibrillary acidic protein GFAP (SP78, Cell Marque,Rocklin, CA, USA, 1 : 100), synaptophysin (27G12, Leica), neurofilament (2F11, Cell Marque, 1 : 100), neuronal nuclear antigen Neu N (A60, Sigma Millipore, Burlington, MA, USA, 1 : 100), P53 (D0-7, Leica), Ki67 (K2, Leica), CD163 (MRQ-26, Cell Marque, 1 : 10), and mutant isocitrate dehydrogenase 1 IDH (R132H, Thermo, (Dianova, Hamburg, Germany), 1 : 20). 

## Fluorescent in situ hybridization FISH analysis for 1p and 19q 

FISH identification of the 1p/19q allelic status was obtained using dual fluorescent-labeled DNA probes (TP73 and GLTSCR). 

## BRAF analysis 

Genomic DNA was isolated from the provided tumor specimen. Exon 15 of the BRAF gene was subjected to SNaPshot multiplex PCR and primer extension for mutation detection. 

## Results 

**Figure 1.**
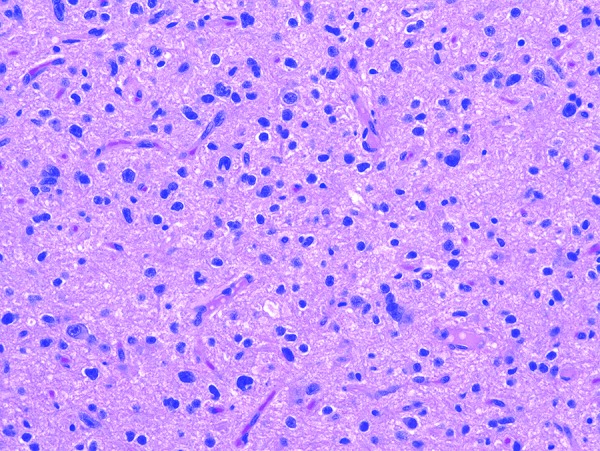
Tumor cells with oligodendroglial-like features (Hematoxylin and eosin H&E stained section, 200 × magnification).

**Figure 2.**
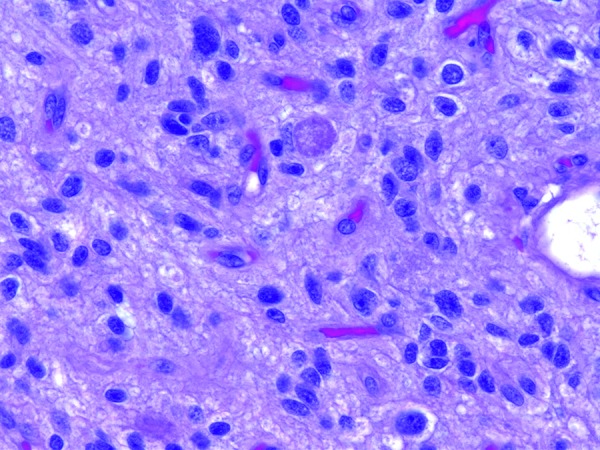
Tumor cells with spindled astrocytic features. Rare eosinophilic granular bodies were noted (H & E stained section, 400 × magnification).

**Figure 3.**
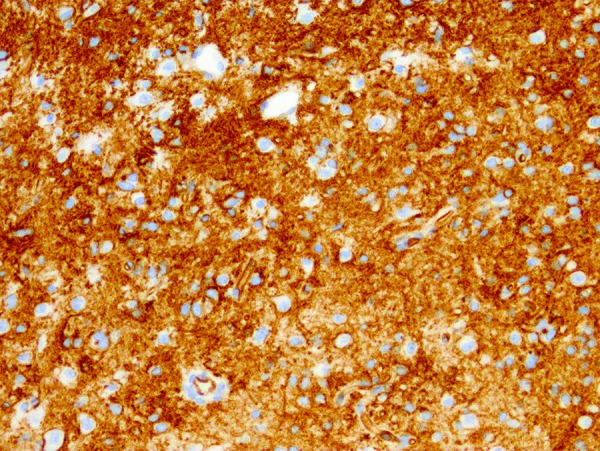
Intense diffuse expression of CD34 in the tumor cells (200 × magnification).

**Figure 4.**
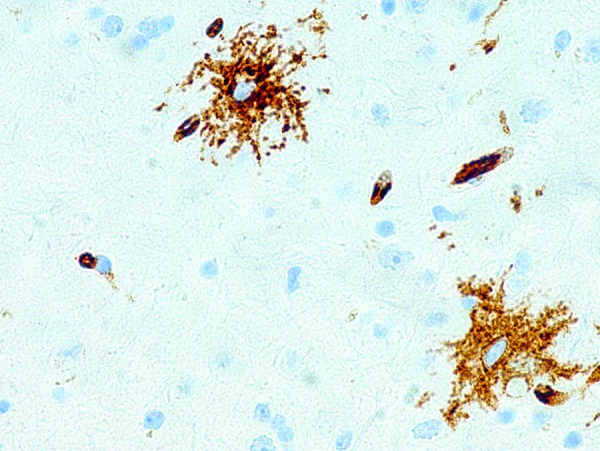
Ramified CD34-expressing neural elements in regional cortex (400 × magnification).

The right temporal lobe specimen showed diffuse but variable hypercellularity of the white matter with clear focal involvement of the overlying cortex ranging from areas with oligodendroglioma-like features containing smaller glial cells with occasional perinuclear halos (Figure 1), to areas with fibrillary or spindled astrocytic features (Figure 2). In spindled areas, one mitotic figure and rare eosinophilic granular bodies were noted. The eosinophilic granular bodies were interpreted as consistent with an astroglial element (Figure 2). GFAP showed patchy, strong cytoplasmic labeling of tumor cells in the astrocytic areas. Many tumor cells exhibited diffuse and intense CD34 expression (Figure 3) with ramified, CD34-expressing neural elements also apparent in regional cortex (Figure 4). Synaptophysin labeled rare neuronal cell bodies likely representing entrapped neurons, while neurofilament positivity was limited to axonal processes and NeuN displayed normal cortical lamination. P53 showed only weak focal positivity. Ki67 labeling index was low (in 1 – 2% range). A mild degree of microgliosis was seen on the CD163. IDH1 (R132H) mutation was negative. Focal to large confluent cortical calcifications were present. 

Chromosomal 1p/19q codeletion was not detected by FISH. BRAF V600E was identified by mutation testing. 

The hippocampus specimen showed cerebral cortex of the temporal dentate gyrus and subjacent white matter. Groups of neurons with no ballooning, or atypical features were noted. Neu N and Neurofilament showed a normal pattern of cortical neuronal bodies and axons. CD34 was negative. GFAP highlighted a mild degree of astrogliosis. 

## Discussion 

Epileptogenic low-grade neuroepithelial tumors encompass a variety of intriguing lesions, which differ from more common central nervous system neoplasms in their clinical context as well as their histopathology. Classification is a rapidly evolving issue in surgical neuropathology, with new entities still being elucidated. 

PLNTY is a low-grade neuroepithelial tumor recently described in a series of 10 patients [5]. In terms of demographic and clinical features, 6 patients were females and 4 were males with an age range of 4 – 32 years at the time of diagnosis. Except for 2 patients, all suffered refractory epilepsy of 1 – 20 years in duration. In terms of location, 7 out of 10 tumors were located in the temporal lobe, and 9 out of 10 lesions were right-sided. Imaging results were available in only 7 cases; all presented as a non-specific unifocal FLAIR hyperintensity with increased or mixed signal on T2-weighted imaging. Neuroradiologic diagnoses included glioma, oligodendroglioma, DNET, and focal cortical dysplasia. Histopathologic variability was observed, tumors harboring, in varying combination, oligodendroglioma-like components, fibrillary/spindled astroglial elements, and ependymoma-like formations. However, all tumors were characterized by the presence of oligodendroglioma-like components, infiltrative growth patterns, and intense CD34 expression by tumor cells. CD34-expressing neural elements were also identified in regional cortex. Calcifications were identified in 9 out of 10 cases and rare mitoses were identified in 2 cases. PLNTs had no 1p/19q codeletion, and genome-wide methylation profiling revealed a distinct signature most closely related to gangliogliomas, with similarities to pilocytic astrocytomas and dysembryoplastic neuroepithelial tumors. Nearly all cases harbored either BRAF V600E mutation or fusion events involving FGFR2/FGFR3. The tumor we report here had similar clinical presentation, demographics, as well as histologic, and immunophenotypic features to the described PLTNY. It also proved BRAF V600E-mutated with absence of 1p/19q codeletion. The status of fusion transcript analysis is unknown in this case. 

Biomolecular profiling studies are becoming increasingly important in characterizing and refining tumor classification. CD34 expression is a common attribute of pediatric gangliogliomas, pleomorphic xanthoastrocytomas, some dysembryoplastic neuroepithelial tumors, glioneuronal microhamartomas, and some cortical dysplasias [5]. BRAF is an important member of the mitogen-activated protein kinase (MAPK) pathway that influences cell proliferation. V600 E being the most common cancer-associated BRAF mutation and one often seen in gangliogliomas, pleomorphic xanthoastrocytomas, dysembryoplastic neuroepithelial tumors as well as a subset of pilocytic astrocytomas [6, 7]. Therefore, the combination of CD34 positivity and BRAF V600E mutation we report raises ganglioglioma, pleomorphic xanthoastrocytoma, and dysembryoplastic neuroepithelial tumor as differential diagnostic considerations. Pilocytic astrocytoma was excluded based primarily on the CD34 expression by tumor cells. The tumor reported lacked defining features of these tumor types and exhibited, instead, features of the PLNTY tumor entity. 

The clinical significance of CD34 expression and BRAF mutation was investigated in a recent publication aiming to study the relationship between biomolecular markers and clinical-pathological features in 22 cases of low-grade epilepsy-associated neuroepithelial tumors [8]. The study found that while CD34 expression (59.1% of cases) was significantly associated with a longer duration of epilepsy (median duration of 16 years), BRAF mutation (50% of cases) was associated with multiple seizure types [8]. It was suggested that BRAF-mutant protein expression might influence neural networks, causing the abnormal discharge of various populations of neurons in different locations, which in turn may contribute to the occurrence of different seizure patterns [8, 9]. Furthermore, the driving molecular alterations, which uniformly activate MAPK signaling in PLNYs, are potentially targetable with existing small molecular inhibitors, providing additional therapeutic options for affected patients [5]. 

Neuroepithelial tumors associated with long-term epilepsy are generally low grade and only exceptionally undergo malignant transformation [10, 11]. Seizure control by surgery may be achievable in as many as 80 – 90% of cases [12]. PLNTYs appear to be typically indolent with excellent seizure outcome after total surgical resection [5]. The patient in this case suffered epilepsy since childhood but recovered following surgical resection of the tumor and is seizure free for 12 months. However, it is generally estimated that it takes more than two decades before an epilepsy patient, including those with low-grade neuroepithelial tumors, is selected to undergo surgery [2]. This is partially due to misclassification of these tumors as non-tumorous lesions. Therefore, early recognition of PLNTY is important to guide clinical management including the indication for surgery. 

## Funding 

None. 

## Conflict of interest 

The authors have no relationships/conditions/circumstances that present a potential conflict of interest.
